# Rapid Classification and Deep Learning-Based Development Estimation of the Seeds of *Helianthus annuus*

**DOI:** 10.3390/plants15131930

**Published:** 2026-06-23

**Authors:** Fami A. Mume, Daniil S. Ulyanov, Temur R. Muratov, Andrey O. Blinkov, Alina A. Kocheshkova, Sergey M. Avdeev, Pavel Yu. Kroupin, Gennady I. Karlov, Mikhail G. Divashuk

**Affiliations:** 1 All-Russia Research Institute of Agricultural Biotechnology, 42 Timiryazevskaya Str., 127550 Moscow, Russiaaoblinkov@gmail.com (A.O.B.); karlov@iab.ac.ru (G.I.K.);; 2 Moscow Center for Advanced Studies, 20 Kulakova Str., 123592 Moscow, Russia

**Keywords:** sunflower, capitulum, seed counting, seed viability, object detection, YOLOv8, phenotyping, computer vision, deep learning, domain adaptation

## Abstract

Manually counting sunflower seeds on capitula is labor-intensive, requiring approximately one person-hour per head, and can be inconsistent for densely packed heads. Existing phenotyping approaches often depend on laboratory-based equipment, limiting their accessibility. In this study, we developed a benchtop image-based pipeline for rapid, non-destructive estimation of developed and aborted seeds on intact dried sunflower heads. A dataset of 1093 sunflower capitula was imaged under fixed indoor lighting, and individual seeds were annotated as developed or aborted. A YOLOv8m one-stage object detector was trained and evaluated using a counting-focused protocol, in which a single confidence threshold was selected on the validation set and then applied unchanged to an independent test set of 109 images. The baseline model was compared with recent YOLO variants and different augmentation strategies. On the test set, the model achieved a mean absolute count error of 61.3 seeds per image, a mean relative error of 12.0%, and an mAP50 of 0.18 at the locked confidence threshold of 0.15. Only 13.8% of test images had relative errors below 2%. Larger YOLO models and augmentation variants did not improve performance. These findings show that the proposed system provides approximate, non-destructive seed-count estimation under controlled imaging conditions, while highlighting the need for improved localization in dense regions and domain adaptation for fresh heads or field conditions. The annotated dataset and trained model weights are made available to support reproducible research.

## 1. Introduction

High-throughput plant phenotyping is a major bottleneck in the improvement of modern crops because reliable phenotypic data are required to link genomic variation with agronomic performance. Despite advances in DNA sequencing that enable rapid, large-scale acquisition of genomic information, the evaluation of plant traits still often depends on labor-intensive, time-consuming manual measurements that are difficult to scale [1,2,3,4]. In the field of plant phenomics, comprehensive assessment of traits related to growth, development, architecture, physiology and yield is therefore essential for breeding programs and crop improvement [2,3,4]. Imaging-based phenotyping offers non-destructive measurement, increased throughput and reduced manual labor; however, extracting reliable biological information from large image datasets remains challenging, especially when target structures are small, densely arranged or partially occluded [1,2,4,5].

Plant image analysis has evolved from manual measurement and rule-based processing toward deep learning-based approaches. Conventional tools such as ImageJ (version 1.45s) and FIJI (release 28 June 2012) improve phenotyping throughput by enabling thresholding, segmentation and morphometric analysis [6]. Nevertheless, these approaches depend on handcrafted features, manual parameter adjustment and dataset-specific thresholds; their performance may therefore vary across imaging conditions, genotypes and developmental stages [5,7]. Deep learning, particularly convolutional neural networks, reduces the need for handcrafted feature extraction by learning image representations directly from raw data [1,4,5,7]. Such methods have been successfully applied to plant identification, disease detection, growth monitoring, yield prediction and other image-based phenotyping tasks [2,3,4,5].

Within deep learning, object detection models are particularly useful for agricultural applications that require both localization and classification of multiple objects in complex images. Object detectors are commonly divided into two-stage models, which first generate candidate regions and then classify them, and one-stage models, which predict object locations and classes in a single forward pass [2,5,8]. Although two-stage detectors can provide high accuracy, one-stage detectors such as You Only Look Once (YOLO) and Single Shot MultiBox Detector are often preferred when inference speed and scalability are important [5,8]. YOLO-based architectures have shown promise in agricultural detection tasks including seed classification and rice panicle detection, yet persistent difficulties remain in detecting small, dense, overlapping or partially occluded objects [8,9,10].

Sunflower (*Helianthus annuus* L.) is an economically important oilseed crop cultivated for oil production, confectionery use and animal feed. Seed yield and seed quality are major breeding objectives; thus, seed number, seed filling and seed abortion are important traits for sunflower breeding and reproductive phenotyping [11,12,13,14]. A single sunflower capitulum may contain hundreds to thousands of florets and seeds arranged in dense spiral patterns, making manual quantification slow, labor-intensive and inconsistent [11,12]. Distinguishing developed seeds from aborted seeds is particularly important because seed development is influenced by genetic, physiological and positional effects within the capitulum. Previous studies have shown that seed and floret size traits are affected by quantitative trait loci and epistatic interactions, while seed position and developmental stage can influence oil content and fatty acid composition [13,14]. Automated detection of developed and aborted seeds on intact capitula could therefore provide useful information for yield estimation and selection.

Earlier studies have used manual or semi-automated image analysis to quantify sunflower reproductive traits. Ochogavía et al. proposed a FIJI-based method for analyzing capitulum images and quantifying reproductive progression, while Sunoj et al. developed digital image-processing approaches for measuring sunflower floral traits [15,16]. Although these methods improve upon fully manual assessment, they still rely on thresholding, manual calibration and controlled imaging conditions, which may limit their scalability across diverse genotypes and environments [15,16].

Deep-learning applications in sunflower seed analysis remain limited. Kurtulmuş applied convolutional neural network architectures including AlexNet, GoogLeNet and ResNet to identify sunflower seed varieties with high classification accuracy, but this approach required harvested, cleaned and manually arranged seeds [11]. Jin et al. developed a multi-objective sunflower seed classification method using YOLOv5 for object detection and ResNet for classification in production-line settings; although this approach handled multiple seeds in video sequences, it also required separated seeds under controlled post-harvest conditions [17]. These studies demonstrate the potential of deep learning for sunflower seed analysis, but do not address the more difficult task of detecting and classifying individual seeds directly on intact sunflower heads.

Despite recent progress, several important gaps remain. Traditional image-processing methods can support sunflower reproductive trait quantification but often require manual calibration and controlled conditions [15,16]. Existing deep-learning methods generally focus on harvested or separated seeds rather than seeds retained on intact capitula [11,17]. In addition, although YOLO-based detectors have been applied to other dense agricultural targets, their use for seed-level detection and developmental-status classification on intact sunflower capitula remains underdeveloped [9,10]. To our knowledge, no published method has yet combined seed-level detection on intact sunflower capitula with automated classification of individual seeds as developed or aborted.

Therefore, the objective of this study was to develop and evaluate a benchtop image-based pipeline for rapid, non-destructive estimation of developed and aborted seeds on intact dried sunflower capitula. Specifically, this study aimed to establish a reproducible seed-level annotated dataset; evaluate a YOLOv8m detector using a counting-focused protocol in which the confidence threshold was selected on the validation set and applied unchanged to an independent test set; report test-set counting accuracy separately from object-detection metrics; compare augmentation strategies and YOLO-family retraining approaches using identical data splits; and define the practical limitations of the method for deployment, generalization and future multi-site validation.

## 2. Materials and Methods

The HARCHOC workflow included image acquisition, preprocessing, seed-level annotation, YOLOv8-based training and inference, held-out test-set evaluation, error analysis and Telegram-based deployment. The system was developed to detect, count and classify developed and aborted sunflower seeds from capitulum images.

### 2.1. Study Aim, Design, Setting, and Statistical Analysis

This study aimed to develop and validate an end-to-end workflow for detecting, counting, and classifying developed versus aborted sunflower seeds from capitulum images. A single-site methodology study was conducted using standardized benchtop image acquisition under fixed indoor lighting on dried sunflower heads. Model development and evaluation were performed using frozen train, validation and test lists comprising 875, 109 and 109 images, respectively.

Statistical analysis prioritized count error on the held-out test set and included standard object-detection metrics at a validation-locked operating threshold. A formal a priori power calculation was not performed because the available CVAT-annotated corpus constrained the sample size, and the evaluation focused on descriptive performance estimation rather than hypothesis testing.

### 2.2. Image Acquisition

**Figure 1:**
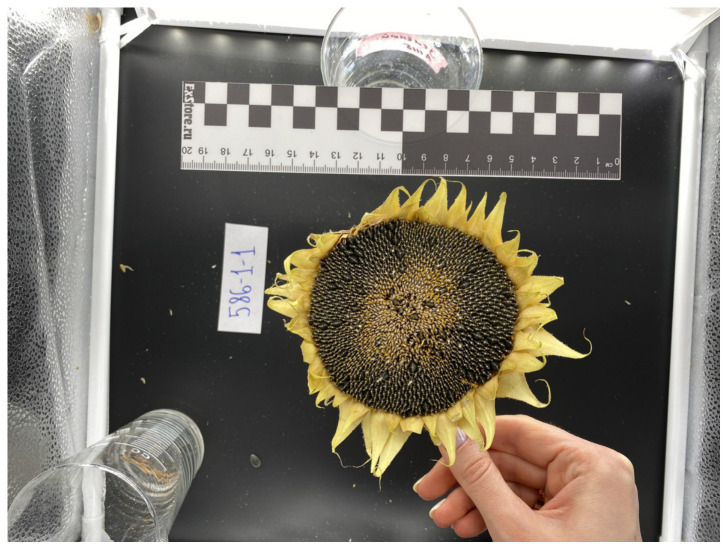
Imaging setup for sunflower head acquisition.

Sunflower capitulum images were acquired under controlled indoor conditions using a fixed benchtop imaging setup, as shown in Figure 1. Images were captured using an iPhone 11 Pro (Foxconn, Zhengzhou, China) mounted above the sunflower head to provide a primarily top-down view. The samples were photographed inside a light-controlled imaging box to reduce external light, shadows and reflections.

Illumination was provided by a Falcon Eyes Light Cube Z40 LED photo studio (Falcon Eyes Ltd., Dongguan, China), which contains two 20 W LED panels, 48 LEDs in total, reflective interior surfaces and a fabric diffuser. Each sunflower head was placed on a fixed support, and a black-and-white ruler was included in the imaging plane as a scale reference. Minor angular adjustments were made when necessary to improve seed visibility, particularly in dense or peripheral regions.

More than 2500 images were initially stored for preprocessing and model development. After curation and annotation, 1093 images were retained for training, validation and testing. Although the imaging protocol provided consistent geometry and illumination, the dataset was limited to single-site indoor imaging and did not fully capture variation from field conditions, different cameras, geographic locations, growth stages or broader cultivar diversity.

### 2.3. Dataset Preparation and Preprocessing

A standardized preprocessing workflow was applied to improve seed visibility and reduce image-to-image variation. The pipeline included contrast enhancement, noise reduction, color normalization, and resizing. Contrast-limited adaptive histogram equalization was applied using a clip limit of 2.0 and a tile grid size of 8 × 8. Gaussian filtering with a 5 × 5 kernel and σ = 1.0 was used to reduce sensor noise. Color normalization was performed by histogram matching to a reference image, and all images were resized to 1500 × 1500 pixels using bicubic interpolation.

Manual seed-level annotation was performed on the preprocessed images using the Computer Vision Annotation Tool. Each visible seed was labeled with an axis-aligned bounding box in YOLO format. Two classes were annotated: developed seed and aborted seed. Developed seeds were defined as full or plump seeds with visible achene development and an intact or clearly developed pericarp. Aborted seeds were defined as flat, empty, poorly developed or collapsed structures showing absent or incomplete achene formation.

Two trained annotators independently annotated the dataset. Disagreements were identified using an automated comparison script and resolved manually during reconciliation. Final consensus annotations were produced according to the agreed operational definitions. Inter-annotator agreement was assessed on 50 randomly selected images and showed strong agreement, with Cohen’s κ = 0.89 and a 95% confidence interval of 0.85–0.93. The final held-out test set contained 109 images, with an average of approximately 554 annotated seed boxes per image.

**Figure 2:**
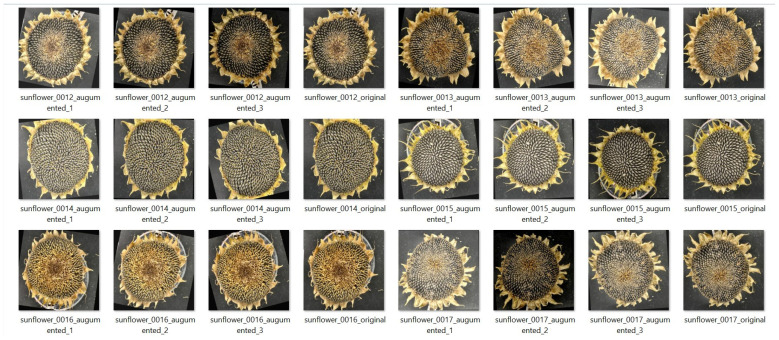
Preprocessed images used for annotation.

Online data augmentation was applied only to the training subset during YOLOv8 training. Augmentation included rotation of approximately ±15°, vertical flipping with a probability of 0.5, four-image mosaic augmentation with a probability of 0.3, brightness adjustment of approximately ±20%, and image scaling. Representative preprocessed images used for annotation are shown in Figure 2.

### 2.4. Annotation Procedure

**Figure 3:**
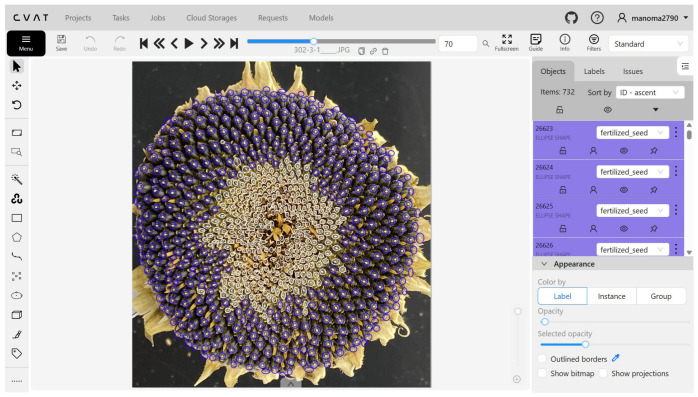
Annotation of image on CVAT.

Annotators manually marked all seeds that were visible in each sunflower head using the Computer Vision Annotation Tool (CVAT, v2.68.0) (Figure 3). Annotators defined two classes of seeds based on success: developed seeds (class 0) and aborted seeds (class 1) (Figure 3). Annotators drew bounding boxes around every individual seed. The bounding boxes cover both clustered seeds and seeds along the head peripheries.

Developed seeds were identified by their full, plump shape, intact pericarp and visible achene development. A flat, empty structure identified aborted seeds with cavities from ovule abortion or absent achene formation. Sunflower breeding specialists helped set this classification threshold. A two-stage annotation system was used for quality control and annotator agreement. Two trained annotators worked independently to annotate all images during the first stage. The automated comparison script ran to detect differences between annotator annotations during the reconciliation phase.

**Figure 4:**
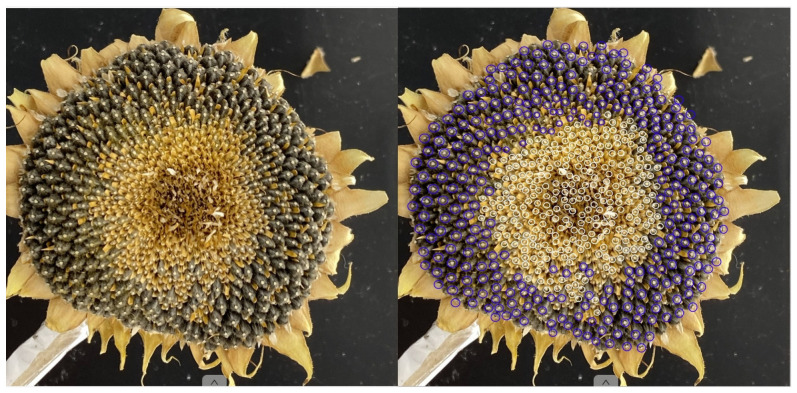
Before and after annotation. Blue bounding boxes show developed seeds, whereas white bounding boxes show aborted seeds.

Consensus labels were established through discussion using operational definitions. Inter-annotator agreement was assessed with Cohen’s kappa on 50 randomly selected images (κ = 0.89, 95% CI: 0.85–0.93). Manual corrections were performed on all annotations to ensure dataset consistency. The final annotated dataset contained 1218 images with 262,346 seed annotations. Of these, 149,430 (56.9%) were labeled as developed, while 112,916 (43.1%) were aborted, resulting in a moderate class imbalance. The training process utilized a class-weighted loss function to address this distribution. Figure 4 illustrates the annotation process by comparing an original sunflower image with the corresponding annotated image showing developed and aborted labels.

### 2.5. Model Architecture

The YOLOv8m (medium) variant demonstrated superior performance compared with other YOLOv8 configurations (YOLOv8n, YOLOv8s, and YOLOv8l) during evaluation on 100 validation images. YOLOv8m delivered an optimal balance between image processing speed and detection precision, running at 28 ms per image on an NVIDIA Tesla V100 (NVIDIA Corporation, Santa Clara, CA, USA) while achieving a test mAP@50 score of 0.180 and a mAP@50–95 of 0.060. YOLOv8m needed less GPU memory than YOLOv8l during operation, using 4.2 GB compared to 8.1 GB. The system operated better on edge devices because of its reduced memory requirements. This configuration matched the validation criteria used for model selection.

**Figure 5:**
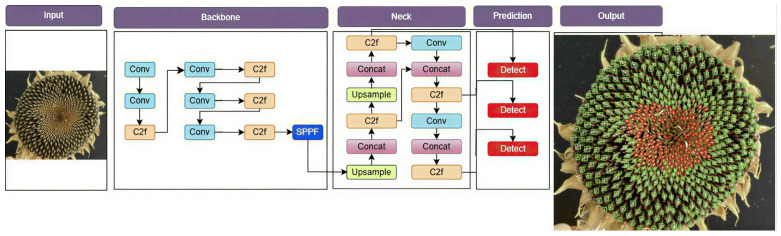
Process of object detection showing the architectural components of YOLOv8m. In the output image, green bounding boxes indicate developed seeds, whereas red bounding boxes indicate aborted seeds.

Figure 5 illustrates the YOLOv8 detection architecture used for sunflower seed detection. The CSP (cross-stage partial) Darnet backbone efficiently extracts features while reducing computational redundancy. The PAN (Path Aggregation Network) FPN neck performs multiscale feature fusion, enabling the detection of seeds across a range of sizes (4–12 mm in images).

The decoupled head separates the classification and localization branches, allowing each to be optimized independently and improving convergence stability.

Anchor-free detection uses center-point regression, improving performance on irregular objects such as seeds and eliminating the need to tune predefined anchor boxes.

The architecture locates individual seeds and their bounding boxes on dense heads. The anchor-free detection method outperforms traditional anchor-based methods because it effectively handles seed irregularities and size differences. The model’s output layer produces two class predictions (developed and aborted seeds), along with seed location information (center x, center y, width, height) and detection confidence values for each detected seed.

### 2.6. Training Configuration

The PyTorch (v2.0.0)-based Ultralytics YOLOv8 framework (v8.0.0) operated on a GPU-equipped workstation to train the model. The validation set served as the testing ground for hyperparameter selection through grid search to achieve an optimal tradeoff between detection accuracy and inference speed. The training process used the following parameters, which were determined through optimization: The final training configuration is as follows:

- Computer hardware: NVIDIA Tesla V100 GPU (32 GB VRAM), Intel Xeon Gold 6248R CPU (3.0 GHz, 24 cores), 128 GB RAM.
- Epochs: 100 training epochs.
- Image size: 1280 × 1280-pixel inputs.
- Batch size: 1 image per batch.
- Optimizer: AdamW.
- Data augmentation: Low mosaic probability (0.1), photometric jitter on HSV channels, random translation and scaling.
- Learning rate: Initial LR (lr0) of 0.0002.
- Max detections: Up to 3000 predictions per image during inference.
- Patience: Early-stopping patience of 50 epochs.

The dataset was split into training (80%), validation (10%) and testing (10%) subsets. The dataset was used for every experiment. YOLOv8 used its augmentation methods. YOLOv8 added flipping with a 0.5 probability. YOLOv8 added rotation ±15°. YOLOv8 added scaling from 0.5 to 1.5 times. YOLOv8 added augmentation with a probability of 0.3.

### 2.7. Evaluation Metrics

The test set, comprising 109 images (10% of the total dataset), was used to evaluate model performance using standard object detection metrics.

- Precision (P): P = TP/(TP + FP), where TP denotes true positives and FP false positives.
- Recall (R): Recall measures the proportion of ground-truth objects successfully detected, calculated as R = TP/(TP + FN), where FN stands for false negatives.
- Mean Average Precision at IoU 0.50 (mAP@50): The evaluation metric mAP@50 calculates average precision at an Intersection over Union threshold of 0.50 to determine single-threshold accuracy. The model generates precision–recall curves for both classes, yielding an average mAP@50.
- Mean Average Precision across IoU thresholds 0.50–0.95 (mAP@50–95): The evaluation metric mAP@50–95 calculates average precision across IoU thresholds from 0.50 to 0.95 at 0.05 increments to assess localization accuracy at different threshold levels.

The evaluation process examined each seed class independently to determine its performance. The model identifies true positives when it detects boxes that match ground-truth boxes in both IoU (≥0.50) and class label. The system identifies false positives when predicted boxes fail to match ground-truth boxes in both IoU value (≥0.50) and class label. The system identifies false negatives when ground-truth boxes do not match any predicted boxes.

The bootstrap resampling procedure (1000 iterations) evaluated the statistical significance of seed-class performance differences using 95% confidence intervals for precision, recall, and F1 scores. The system produced confusion matrices to display seed-class prediction errors across different seed types.

### 2.8. Model Validation and Error Analysis

For an additional manual verification analysis, 50 images were randomly selected from the held-out test set. The subset was chosen to represent the range of seed densities observed across the full test dataset, including low, medium, and high-density capitula. An expert annotator, blinded to model predictions, independently counted seeds on these images. The resulting counts were compared with automated predictions to assess counting accuracy and characterize major error sources.

The counting accuracy calculation used the following formula:

$$Relative\;Error\;\left(\%\right)=\;\left|\frac{{AI}_{count}-{Manual}_{count}}{{Manual}_{count}}\right|\;\times 100$$

The analysis of errors focused on images with a relative error exceeding 15%. The review of failed images revealed four main error sources: seed occlusion and overlap; seeds near developmental boundaries; and imaging defects caused by glare and shadows.

### 2.9. Model Deployment via Telegram Bot Interface

In HARCHOC, the trained YOLOv8 model was deployed via a Telegram bot interface that accepts sunflower images as photo or document uploads. This two-stage pipeline (content verification followed by seed detection) is conceptually similar to the two-stage detection pipeline (person detection followed by identification) used in [18] for UAV-based multi-person detection. For valid images, a detection pipeline using SAHI (Slicing Aided Hyper Inference, v0.11.18) applies an Ultralytics YOLO model in a sliding-window fashion, with a configurable slice size (e.g., 800 × 800 pixels) and an overlap ratio (0.15) to accurately localize small seeds. Detected objects are post-processed using non-maximum suppression (NMS), and each seed is classified as developed or aborted based on model confidence thresholds (default 0.12). Counts and development percentages are computed, and bounding boxes (green for developed, red for aborted) are drawn on the original image without text labels. The annotated image and textual results are returned to the user via Telegram. The system supports GPU acceleration (CUDA, v13.2) for inference speed, includes retry logic for network timeouts, and runs a health-check server for deployment on platforms like Railway.

- Input image: Users upload images through the bot.
- Model inference: The YOLOv8 model performed seed detection and classification through inference with a confidence threshold of 0.772 (the F1-optimal threshold identified from the F1–confidence curve) and Non-Maximum Suppression IoU threshold of 0.45.
- Results reporting: The system generates output and provides a summary report that shows total seed numbers and developed seed numbers.

The total end-to-end processing time per submitted image via the Telegram bot ranged from 15 to 30 s, including image upload, server-side inference (112 ms on the GPU), result rendering, and message delivery to the user. This response time was acceptable for real-time field-based phenotyping during breeding activity.

The developed system is available as a Telegram bot [19] and the source code is available on GitHub [20]. The CVAT annotated dataset is available via a public share link.

## 3. Results

### 3.1. Detection Example and Operating Point

**Figure 6:**
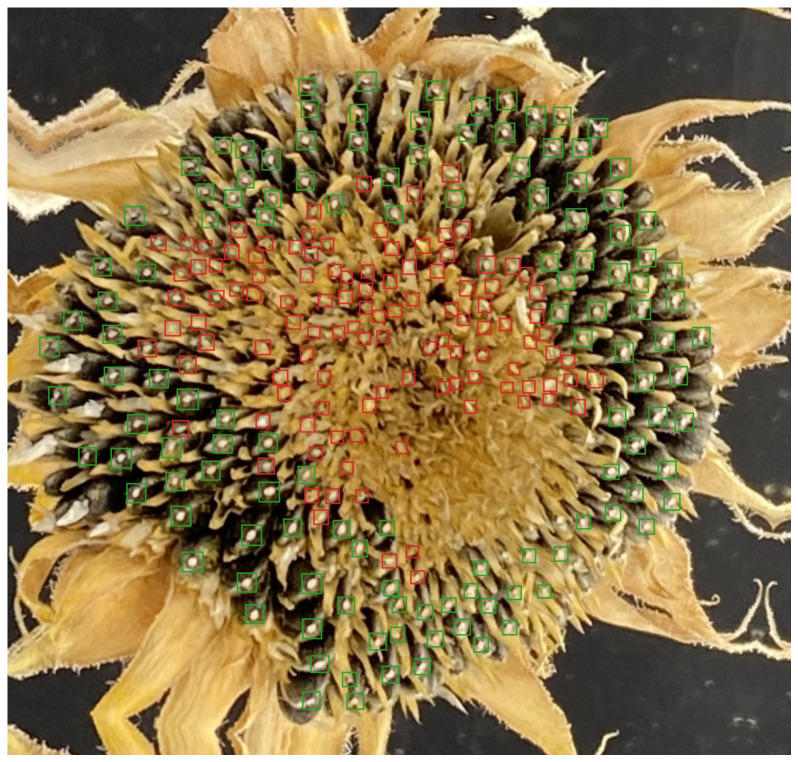
Representative HARCHOC detection output for developed and aborted sunflower seeds. Green bounding boxes indicate developed seeds, whereas red bounding boxes indicate aborted seeds.

HARCHOC localizes individual sunflower seeds and assigns each instance to developed or aborted classes. Figure 1 shows a representative detection output generated from the updated reproduction package. For manuscript metrics, predictions were exported at low confidence and evaluated under a validation-locked operating confidence of approximately 0.15; this operating point was selected to minimize count MAE rather than to maximize F1. Figure 6 illustrates the model output at the selected operating point, showing the localization and classification of individual sunflower seeds.

### 3.2. Model Training and Convergence Analysis

The production detector was retained from the public sunflower-Detector checkpoint and re-evaluated with the frozen HSP counting protocol. Comparative 100-epoch retraining experiments were run on identical splits to test whether a new YOLO-family training recipe surpassed the production anchor.

**Figure 7:**
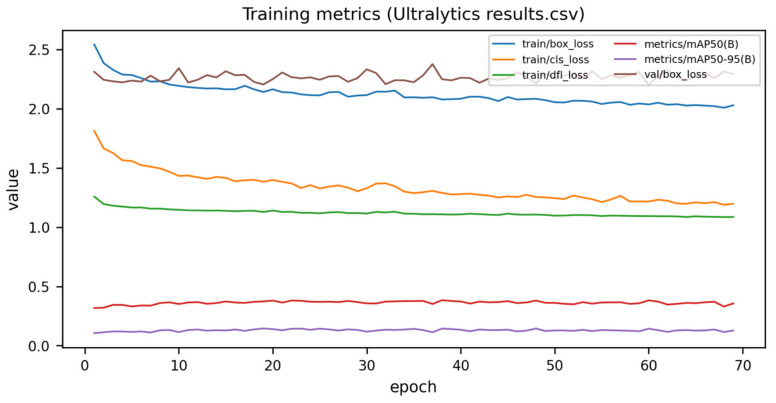
Training and validation curves used for reproduction and convergence checks.

Training and validation curves (Figure 7) are reported for transparency, but peak validation mAP during training is not used as a generalization claim. Held-out test count MAE at the validation-locked operating confidence is the primary endpoint for model comparison.

All quantitative results below use the same frozen test split and the same locked confidence selected on the validation split. No test image influenced threshold selection.

### 3.3. Corpus, Splits, and Density

**Figure 8:**
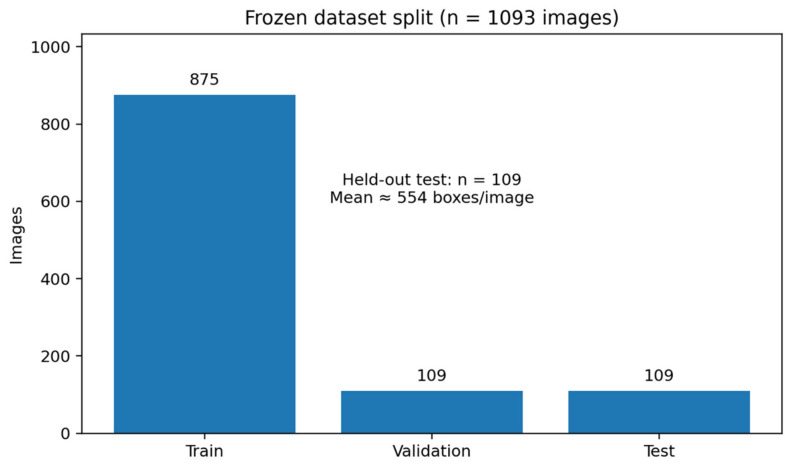
Frozen corpus split used for the updated HSP evaluation. The held-out test set contains 109 images with a mean density of approximately 554 ground-truth boxes per image.

The updated CVAT-annotated corpus contains 1093 dried sunflower capitulum images acquired under fixed benchtop lighting at a single site. Figure 8 presents the frozen dataset split used for model development and evaluation. Frozen train, validation, and test lists contain 875, 109, and 109 images, respectively. Each visible seed was annotated with an axis-aligned bounding box and assigned to the developed or aborted class.

The evaluation therefore reflects dense benchtop seed counting under controlled acquisition conditions. It does not establish field robustness across natural lighting, fresh heads, multiple varieties or geographic sites.

### 3.4. Primary Detection and Counting Performance

Table 1 summarizes the updated object-detection and counting metrics from the reproduction package. The primary generalization metric is held-out test count MAE at the validation-locked confidence.

On the held-out test split, the production anchor achieved a count MAE of 61.3 seeds per image. The validation row is reported only to document the threshold-selection step, it is not a substitute for test-set performance.

Detection ranking metrics are reported separately from count error. At the locked operating point, test mAP50 was 0.180 and mAP50-95 was 0.060, reflecting the difficulty of dense localization and duplicate/background errors.

Because breeding use depends on seed counts, all augmentation and model-zoo comparisons were ranked by held-out test count MAE rather than by validation mAP or a single F1-optimal confidence.

### 3.5. Confusion Matrix and Error Taxonomy

**Figure 9:**
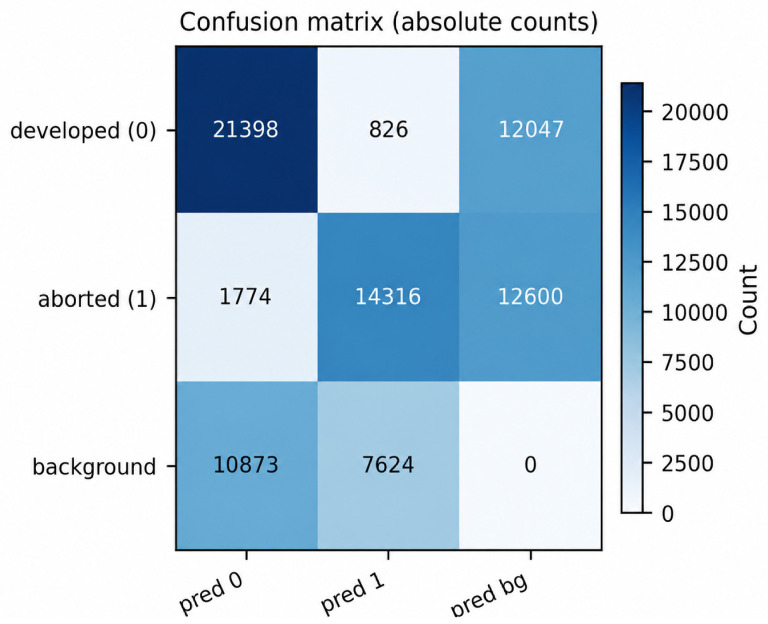
Absolute confusion matrix for the updated held-out evaluation.

Figure 9 presents the absolute confusion matrix from the updated evaluation export. In dense seed scenes, most practical errors are due to missed and duplicate detections, as well as background-associated detections, rather than simple cross-class swaps between developed and aborted seeds.

**Figure 10:**
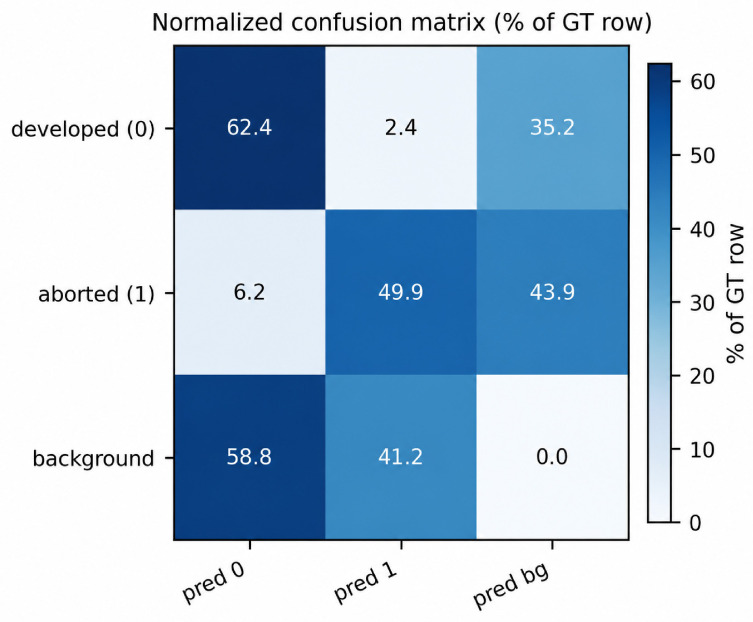
Normalized confusion matrix for developed and aborted seed detection.

Figure 10 presents the normalized confusion matrix. Normalized proportions help separate class-confusion patterns from error volume and support comparison across dense heads with different seed counts.

The remaining errors are consistent with dense small-object detection: central overlap, seed boundary ambiguity, partial occlusion, glare or shadow, and near-background structures that resemble seeds.

**Figure 11:**
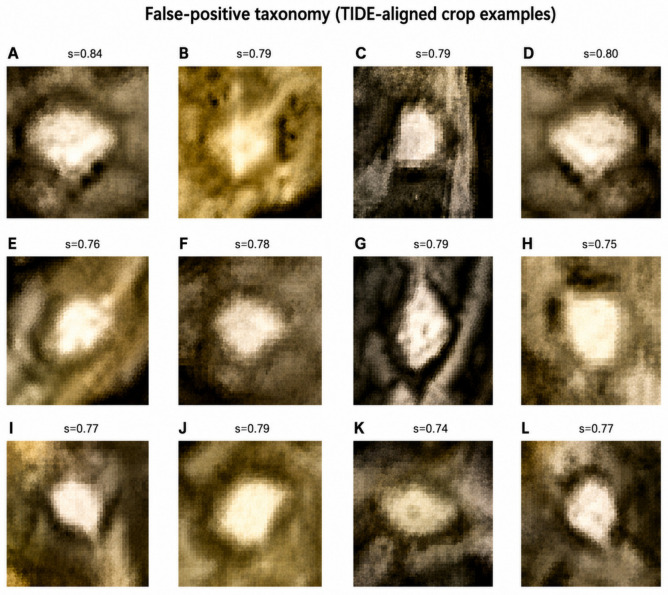
Error taxonomy visualization showing the distribution of false positive sources. Panels (**A**,**D**,**G**,**J**) correspond to background errors; panels (**B**,**E**,**H**,**K**) correspond to classification errors; and panels (**C**,**F**,**I**,**L**) correspond to localization errors. The values above each panel indicate the associated confidence scores.

Error taxonomy analysis (Figure 11) revealed the dominant error modes: missed detections accounted for 52.9% of all errors (32,884 instances), localization errors comprised 27.5% (17,107 instances), background false positives contributed 16.7% (10,402 instances), and class confusion was minimal at 2.9% (1825 instances). This pattern confirms that the primary challenge in dense sunflower seed detection is complete misses and localization inaccuracy rather than confusion between seed classes.

### 3.6. Precision–Recall and Confidence-Threshold Analysis

**Figure 12:**
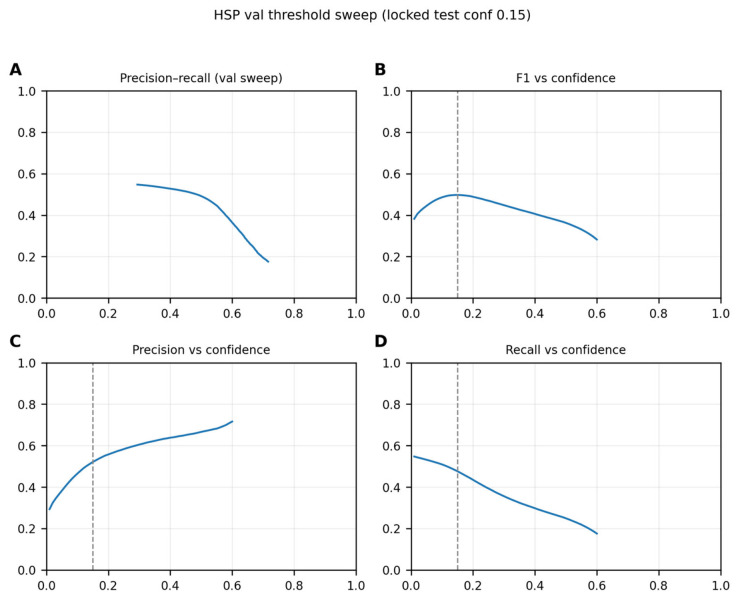
(**A**–**D**) Updated precision–recall, F1–confidence, precision–confidence, and recall–confidence diagnostics.

Figure 12 summarizes precision, recall, F1, and precision–recall behavior under the updated reproduction. These curves are retained for diagnostic interpretation, but the selected operating threshold is the validation-locked count-MAE threshold.

The key methodological correction is that the deployment-style F1 threshold and the training-validation peak mAP must not be used as the manuscript’s generalization metric. The held-out test metrics in Table 1 define the reported performance.

The locked confidence of approximately 0.15 was selected on validation images by minimizing total count MAE and then applied unchanged to the test split.

This threshold favors count recovery in dense scenes and may allow more low-confidence detections than an F1-optimized threshold, which is why count error and ranking mAP are interpreted separately.

### 3.7. Full-Test Counting Validation

Table 2 reports full held-out test counting error at the validation-locked confidence. The full *n* = 109 test distribution is the manuscript’s headline counting-validation set.

The held-out test set had a mean of approximately 554 ground-truth boxes per image. Mean relative error was 12.0%, median relative error was 9.3%, and 13.8% of images had relative error below 2%.

Table 3 shows that most test images fall between 5% and 20% relative count error, with a smaller low-error subset and a high-error tail driven by dense overlap and image-quality artifacts.

## 4. Discussion

### 4.1. Counting-First Performance in Context

The updated HSP reproduction reframes HARCHOC as a counting-first benchtop phenotyping workflow. On the held-out test set, the production anchor reached a count MAE of 61.3 seeds per image with a 95% bootstrap confidence interval of 51.3–71.3. This value is the primary performance claim because the breeding task requires accurate seed counts, not only high-ranking detection curves.

Dense sunflower capitula differ from many plant-detection tasks because hundreds of small, touching instances can appear in a single image. Evaluation therefore needs to distinguish count recovery from localization stringency, especially when overlapping seeds generate duplicate and background-associated errors.

### 4.2. Detection Ranking Versus Count Accuracy

The updated test mAP50 was 0.180 and mAP50-95 was 0.060 under the canonical evaluation path. These ranking metrics are lower than the count-level performance and should be interpreted as localization diagnostics rather than as the headline breeding endpoint.

The validation-locked count-MAE threshold intentionally prioritizes count recovery in dense scenes. A higher F1-optimized threshold can produce cleaner detections but may undercount many seeds. Therefore, the manuscript reports count MAE, relative error, and ranking mAP as separate quantities.

### 4.3. Comparative Augmentation and Model-Zoo Results

The updated reproduction adds a SOTA-response comparison across augmentation ablations and YOLO-family retrains. A 100-epoch augmentation confirmatory run achieved MAE 64.1, close to but not better than the production anchor. Completed YOLO-family retrains on the same splits also did not beat the anchor: YOLOv8m retrain MAE 111.9, YOLO11m MAE 119.6, and YOLO26m MAE 95.3. These results support retaining the production checkpoint while presenting the comparison as evidence of robustness rather than algorithmic novelty [21,22,23].

### 4.4. Error Sources

The main residual errors are biological and imaging ambiguities: severe central overlap, partially visible seeds, near-boundary developed versus aborted morphology, glare or shadow, and background textures that resemble seed edges. These errors explain the long tail in Table 3 and are consistent with dense small-object benchmarking challenges.

In addition to dense overlap and occlusion, performance may be affected by non-uniform seed distributions across the capitulum. Most images in the present dataset contained relatively typical sunflower head morphology; however, some capitula exhibited asymmetric seed filling, irregular abortion patterns, or localized regions with sparse seed density. Such spatial heterogeneity can increase both missed detections and localization errors because the detector was primarily optimized using the dominant distribution patterns present in the training set. Future work should evaluate performance separately on atypical capitula with highly uneven seed distributions and investigate spatially aware detection strategies that better accommodate heterogeneous seed arrangements.

Future error analysis should keep count error, duplicate detections, missed detections, and cross-class confusion as separate categories. Ambiguous seed-development boundaries may also benefit from calibrated uncertainty or graded decision rules rather than a forced binary decision [22].

### 4.5. Practical Deployment Boundaries

The Telegram bot demonstrates an accessible user interface for image submission and result reporting, but deployment logs were not part of the updated reproduction bundle. Therefore latency, uptime, and operational success-rate claims are not used as scientific performance metrics in this manuscript.

Manual counting and separation of developed versus aborted seeds remain labor-intensive in breeding practice. HARCHOC is most defensible as a high-throughput screening aid under controlled benchtop imaging, with manual review retained for outlier images and downstream breeding decisions.

### 4.6. Implications for Genomic Selection

Automated counting can increase throughput for phenotyping pipelines when the measurement protocol is fixed and quality-control rules are explicit. Total seed count and developed-to-aborted ratio provide complementary traits for selection, but the current single-site dataset should not be treated as a general field model without additional validation.

### 4.7. Generalization and Future Directions

All headline data were acquired from dried heads under controlled indoor lighting at one site. Field lighting, fresh material, other camera systems, multiple varieties, and cross-regional conditions remain untested. Following the transfer learning approach of [24], we plan to pre-train on our current dataset and fine-tune on a small set (50–100 labelled images per new environment) of fresh heads under field lighting or with other camera sensors. This domain adaptation pathway offers a practical route to broader generalization. work should add multi-site data, report tray/session variability, and evaluate domain adaptation rather than assuming direct transfer [25,26,27,28].

Future extensions may include instance segmentation, calibrated uncertainty for ambiguous seeds, explainable heatmaps for reviewer-facing diagnostics, and a prospective deployment audit that reports latency, failure modes, and user-facing thresholds separately from the scientific benchmark. Additionally will explore three-dimensional reconstruction, hyperspectral imaging fusion, and instance segmentation to broaden phenotypic assessment; field-based detection from UAV imagery represents another logical extension [29]. To improve trust in breeding programs, future work will adopt interpretable AI methods, such as those in [30], to visualize which image features (seed color, texture, size) drive the classification of developed vs. aborted seeds.

### 4.8. Generative AI Use Statement

The authors used DeepSeek-V4 solely for grammar and style refinement of the final manuscript draft. The initial scientific content, data analysis, figures, and all critical interpretations were produced without the use of generative AI. All AI-assisted edits were manually reviewed and fact-checked. Cursor IDE (v3.6.31) was extensively used during model development.

## 5. Conclusions

HARCHOC provides a reproducible benchtop workflow for detecting, counting, and classifying developed versus aborted sunflower seeds on dried capitula. Under the updated frozen HSP evaluation, the production YOLOv8m anchor achieved held-out test count MAE of 61.3 seeds per image (95% CI: 51.3–71.3), mean relative counting error of 12.0%, median relative error of 9.3%, and test mAP50 of 0.180 at a validation-locked confidence of approximately 0.15. Completed YOLO-family retrains and augmentation comparisons did not surpass the production anchor on test count MAE. The system is best interpreted as a controlled benchtop phenotyping aid; field imaging, fresh material, multi-variety and multi-site validation, and audited deployment logs remain necessary before broader operational claims are made.

## Figures and Tables

**Table 1:** Object detection and counting metrics under the frozen HSP reproduction protocol.

Split	Count MAE	95% CI	mAP50	mAP50-95	Conf.
Test	61.3	51.3–71.3	0.180	0.060	0.15
Validation	71.0	58.8–84.2	—	—	0.15

**Table 2:** Counting summary on the held-out test split.

Metric	Value
Locked confidence	0.15
Test images (*n*)	109
Count MAE	61.3
Mean relative error (%)	12.04
Median relative error (%)	9.34
Images with relative error < 2% (%)	13.8

**Table 3:** Distribution of relative counting error on the held-out test split.

Relative Error Bin	Images (*n*)	% of Test
<2%	15	13.8
2–5%	19	17.4
5–10%	24	22.0
10–20%	38	34.9
>20%	13	11.9

## Data Availability

The annotated dataset, trained model weights, and associated manifests are publicly available in the project repository at [20]. All data required to reproduce the analyses reported in this study are already available at the time of publication.

## Abbreviations

The following abbreviations are used in this manuscript:

AI	Artificial Intelligence
CLAHE	Contrast-limited adaptive histogram equalization
CNN	Convolutional Neural Network
CVAT	Computer Vision Annotation Tool
DFL	Distribution Focal Loss
F1	Harmonic mean of precision and recall
FN	False Negative
FP	False Positive
IoU	Intersection Over Union
mAP	Mean Average Precision
NMS	Non-Maximum Suppression
PAN	Path Aggregation Network
PR	Precision–Recall
SGD	Stochastic Gradient Descent
TP	True Positive
YOLO	You Only Look Once
